# Coping strategies of mothers with preterm babies admitted in a public hospital in Cape Town

**DOI:** 10.4102/curationis.v42i1.1872

**Published:** 2019-10-01

**Authors:** Delphine A. Sih, Million Bimerew, Regis R.M. Modeste

**Affiliations:** 1 Department of Nursing, University of the Western Cape, Cape Town, South Africa; 2 Department of Nursing Science, Cape Peninsula University of Technology, Cape Town, South Africa

**Keywords:** coping strategies, preterm babies, neonatal care unit, preterm delivery, pregnancy, traumatic experience

## Abstract

**Background:**

Most pregnancies run a normal course, ending in a healthy mother–infant relationship, but sometimes, it can also be a life-threatening and stressful condition. The stress levels of mothers are more aggravated when they deliver preterm babies.

**Objectives:**

To explore the coping strategies of mothers of preterm babies with the stress of preterm delivery and subsequent admission of the preterm neonate to a neonatal care unit.

**Method:**

A qualitative research approach applying an exploratory and descriptive design was applied to explore the coping strategies of mothers with preterm babies admitted in a neonatal care unit. The study applied a purposive sampling technique to select mothers with preterm babies. The population for this study included women who delivered preterm babies and whose babies were admitted in the neonatal care unit at a public hospital in Cape Town. Semi-structured interviews were conducted until data saturation was reached, and 11 mothers with preterm babies in the selected public hospital participated in the study. Data were analysed manually using thematic content analysis with an inductive approach.

**Results:**

Results were deductively interpreted and supported by the Brief COPE model. The main themes that emerged from data analysis included praying, attachment with baby and acceptance of the situation. Under praying the following subthemes emerged, namely praying for God’s strength, God’s grace, babies’ survival and thanksgiving to God for babies’ health and preferred gender. The theme of attachment with the baby emerged with the following subthemes: bonding with the baby and seeing the baby. The last theme that was acceptance of the situation emerged with the following subtheme: perseverance in the situation and mother’s awareness of her responsibility.

**Conclusion:**

Even though the mothers of preterm babies cope differently after delivery, their coping abilities, which included praying, attachment to baby and acceptance of the situation, were greatly determined by the condition of their babies as well as the support they receive from significant others.

## Background information

Most pregnancies run a normal course, ending in a healthy mother–infant relationship. However, the rate of preterm delivery is shown to be increasing worldwide, and grew from about 9.6% of all babies born preterm in 2005 (Beck et al. 2010) to about 10% in 2010 (World Health Organization [WHO] 2012). Preterm delivery can be challenging and affects the mothers, their babies and other family members (WHO 2012). According to the WHO (2012), preterm delivery is defined as all deliveries that occur before 37 completed weeks of gestation. It was identified that based on the gestational age and birth weight, preterm delivery can be categorised as extremely preterm, very preterm and moderately preterm (WHO 2012).

According to the studies conducted by Raju et al. (2014) and Woodward et al. (2014), survival of preterm babies often depend on important aspects related to the mother as well as the health system. Aspects, such as mother–baby bonding, mothers’ coping ability, availability of appropriate equipment for neonatal care and competent neonatal care personnel, have been mentioned to have supportive care to both mothers and their neonates throughout the period of admission (Hoffenkamp et al. 2012; Lloyd & De Witt 2013; WHO 2012). A similar study identified that positive coping is beneficial to the health of mothers and their babies (Wu et al. 2013). The literature has also noted that some mothers of preterm babies use negative coping strategies after preterm delivery (Ghorbani et al. 2014; Shaw et al. 2013). It is important to note that non-effective coping strategies have been associated with negative outcomes (Dardas & Ahmad 2015). Health care professionals play a vital role to encourage positive coping and to do this, mothers’ individual coping strategies need to be understood by health care professionals tasked with the responsibility to care for mothers of the neonatal care unit.

## Problem statement

Mothers with preterm babies admitted in a neonatal care unit experience stress and would need to adapt to the changing state of responsibility in their lives. To do so, these mothers have to develop effective coping strategies in order to deal with the stressful situation (Shosha & Kalaldeh 2012). Even though preterm babies are usually admitted for crucial hemodynamic monitoring and special health care, mothers of these babies often face several daunting challenges, such as interrupted mother–baby bonding, depression, anxiety and fear of losing their babies (Kendall-Tackett 2009; Van der Hoeven, Kruger & Greeff 2012). Moreover, preterm delivery can negatively affect mothers by reducing maternal self-belief and maternal confidence. Preterm delivery can also cause mothers to feel helpless, which can eventually lead to inability of the mother to cope and subsequently inability to care for her preterm baby (Korja, Latva & Lehtonen 2012). Hence, preterm babies run the risk of poor growth and development because of poor attachment in mother–baby bonding. Considering that effective coping strategies are linked to positive outcomes while ineffective coping strategies are linked to negative outcomes, it is important to understand coping strategies used by mothers of admitted preterm neonates, as the well-being of the neonates can be affected by the mothers’ coping strategies during the neonates’ admission (Dardas & Ahmad 2015). To date, little is known about the coping strategies of mothers with preterm babies in the neonatal care units in Cape Town. This research hoped to explore coping strategies used by mothers of preterm babies, while their preterm babies are admitted in the neonatal care unit in a public hospital in Cape Town.

## Research objective

The objective of the study was to explore and describe the coping strategies used by mothers whose preterm babies were admitted in a neonatal care unit in a public hospital in Cape Town.

## Research method and design

A qualitative research approach and an exploratory and descriptive design were utilised in this study. Information regarding mothers’ coping strategies was acquired through interviews and probes which allowed sufficient exploration and gaining of insight to the coping strategies used by mothers after delivering a preterm baby. Data were gathered using semi-structured interviews guided by the Brief COPE model. The aspects of the Brief COPE model were used to interpret the information that was collected from participants of this study. The Brief COPE model was chosen because it is designed to assess coping in a naturally occurring environment. Mothers were interviewed at the environment where their babies were admitted. Only English and Xhosa speaking mothers older than 18 years participated in this study.

## Sampling and sample size

The population for this study consisted of women whose preterm neonates were admitted in the neonatal care unit at the public hospital in Cape Town. Through purposive sampling technique, mothers whose preterm babies were admitted to a neonatal care unit at public hospital in Cape Town were recruited. The inclusion criteria were being 18 years and older, and having a preterm baby admitted in the hospital at the time of interview. This fits the assertion by Roberts (2014), who indicated that at this age, the mothers are legally fit to consent for any action in general, and for participation in research as in this case. Twelve mothers who fulfilled the inclusion criteria were approached and given an information letter by nurses working in the neonatal care unit to participate in the study, but only 11 mothers consented to participate. Data saturation was noted at the ninth interview and interviews continued with two more participants to confirm that no new information was coming up.

## Data collection method

Participants who accepted to participate were given the opportunity to choose a convenient date and time to conduct the interview. Semi-structure interviews were conducted with the participants, each lasting about 40–45 min. One main question was asked in the interview, and probes were used to gain in-depth understanding of the phenomenon. As most participants preferred English language, the researcher conducted the interviews. An experienced qualitative data collector who is fluent in Xhosa language was used to collect data from one participant who preferred Xhosa language for the interview. It was agreed that the trained data collector would be called in should there be a participant with Xhosa or Afrikaans language preference rather than English language. The information sheet was explained and informed consent was obtained before commencing each interview, allowing the researcher to ensure that the participants understood the purpose of the research, as well as the implication of participation. After participants’ permission, interviews were audio-recorded after which the researcher transcribed verbatim and qualitatively analysed the collected data.

## Data analysis

Fitting the qualitative approach adopted in this study, data collection and data analysis were performed concurrently. An inductive thematic content manual data analysis was executed. All audio-recorded interviews were transcribed verbatim. The Xhosa transcript was translated into English, and translated back into Xhosa by a professional Xhosa language translator to ensure that the translated interview carried the same meaning. The inductive data analysis approach (De Vos et al. 2011) was followed as: All interview transcripts were labelled and transcribed verbatim in a chronological manner. Preliminary coding was performed reading through the transcripts and similar codes were identified. Main themes and subthemes were obtained. The themes were described one after another and for each theme, a quote from a participant’s words was mentioned. The coded materials were then interpreted deductively by comparing with the coping aspects of the established Brief COPE model.

## Trustworthiness

Criteria of credibility, transferability, dependability and confirmability were used to maintain trustworthiness in this qualitative research study (Elo et al. 2014). Credibility was ensured through member check, by returning findings to participants to determine if the findings reflect their accounts. With this practice, the researcher had the opportunity to resolve any unclear issue that could have been identified during data transcription and analysis. Prolonged engagement with participants during the data collection period provided the opportunity for the researcher to interact with participants and ensure credibility.

Transferability was ensured in this study by giving a clear description of the setting where this research was based. The research process has been sufficiently described to ensure a broader understanding that can be used to have an understanding of a similar context.

To ensure dependability of the study, an independent coder was used to ensure consistency in the coding process. Again, an audit trail was maintained in this research. Each step of this research process was described in full and the research report was independently reviewed by a peer reviewer. In this research, confirmability was ensured by the use of audit trials to describe the detailed process of data collection, analysis and field notes during data collection and interpretation of the data. The verbatim transcripts as well as the audio-taped records were given to the independent coder, who was able to establish consistency in the coding process.

## Ethical considerations

Trustworthiness of the study was maintained through credibility, confirmability, dependability and transferability. Ethics approval to use human subjects for study purposes was obtained from ethics committee of the University of the Western Cape (permission number: 15/6/7), and permission to use the health care facility for the study was granted by the institutional authorities and from the neonatal care unit manager. The research participants were recruited and they volunteered to take part in the research, and consent was obtained. The participants were informed that they can withdraw from participating in the study at any time without any consequences. The ethical principles that guided this research included beneficence, autonomy, respect and justice.

To ensure autonomy in this research, participants were not forced to participate but were given the opportunity to decide to do so. The participants were informed in advance about the scope of this research, including the potential benefits and risk of their participation. The information sheet was handed to participants before interviews took place. Participants were again clarified about the research aim and objectives just before commencement of the interview and a consent form was obtained from participants before the interview. The principle of respect was maintained through voluntary participation as well as the freedom to withdraw at any stage of the research process without any consequences.

To maintain the principle of justice in this research, every mother who was identified was given a fair chance to take part in this research. Volunteers who met the inclusion criteria were given an introduction to the study. The researcher informed participants that the interviews were to be audio-recorded, and permission for this was obtained individually from every participant. All recordings and transcripts remained stored and locked in a cupboard in the researcher’s room and the recordings were kept safely. No identifiable information was attached to the data that were collected and code was used instead. To be able to adhere to the beneficence principle, harm was prevented and participants’ well-being was ensured, with plans being made for further support and counselling in case of any experience of stress and discomfort. However, none of the participants reported any negative effect from their participation in the study.

## Results

The major themes that emerged revealed that mothers of preterm babies coped through praying, attachment to baby and acceptance of the situation. Under the main themes, emerged subthemes are listed in Table 1.

**TABLE 1:** Themes and subthemes emerged.

Theme	Subthemes
1. Praying as coping strategy	1.1. Thanksgiving to God for babies’ health and preferred gender. 1.2. Praying for God’s strength, God’s grace, babies’ survival.
2. Attachment to baby	2.1. Bonding with baby and seeing the baby. 2.2. Evidence of life, progress on babies and no regrets.
3. Acceptance of the situation	3.1. Perseverance in the situation. 3.2. Mother’s awareness of her responsibility.

## Theme 1: Praying as coping strategy

Praying was one of the themes that emerged which most participants mentioned as a coping strategy. Most of the participants mentioned that practising their spiritual beliefs involved praying. Two subthemes emerged from the main theme of praying. The two subthemes were praying for God’s strength, God’s grace, babies’ survival and thanksgiving to God for babies’ health and preferred gender. As one participant put it:

‘I pray a lot every time I go visit him, I pray and when I leave, I pray because … In the evening I pray.’ (P4, female, 23 years old)

### Subtheme 1: Thanksgiving for babies’ health and preferred gender

Participants expressed being grateful for the lives of their babies as well as for being able to experience motherhood irrespective of the state in which their baby was born as one mother mentioned:

‘Ok. Everything about her, the way she looks at me. That is stuff it keeps me going. That is why I am not regretting the way she was born small.’ (P11, female, 20 years old)

As one participant stated, she expressed thankfulness to God for giving her a healthy baby.

‘I can thank God for giving me such a little baby and she is growing well and everything is going good for her, even for other mommies, I wish them all the luck and if they know what the baby needs is the blessings from God, and I appreciate my blessing that I have from God.’ (P11, female, 20 years old)

Participants also expressed thanks to God for the baby’s gender. This was illustrated by some of the participants who expressed gratitude for a preferred gender for a male or female child as a result of their pregnancy. One participant particularly thanked God for giving her a female baby and mentioned that:

‘I feel better that the Lord give me another child who is a girl so the boys have a sister [*laughing*].’ (P7, female, 33 years old)

### Subtheme 2: Praying for God’s strength and grace and baby’s survival

Praying for God’s strength, grace and babies’ survival could refer to asking God for the power to withstand the pressure caused by a specific event. This is illustrated by the one participant who stated that:

‘Basically I pray to God for strength so that I can get through the day.’ (P6, female, 21 years old)

Preterm delivery may tempt mothers to act negatively towards their baby as one participant stated that:

‘In the meantime I think I’ll leave my child alone or something like that but I said no! That the Lord is going to help me. And I’ll be strong.’ (P7, female, 33 years old)

Participants prayed for the grace of God to be conferred upon them and help them in their time of need.

One said that:

‘I just feel sad because why, I want to go home. I miss my other children also … I can’t go because of this child “mos” [*because*] I must give breastfeeding. But I just hope the Lord come and help me through this.’ (P5, female, 29 years old)

Participants prayed excessively for the survival of their babies and one mentioned that:

‘Because when lay in Hospital X, I did see many children who were dying. It’s dead, normal birth, Caesar cuts, the babies die, so I think yoh! It can happen to me, so I did, I did, pray, pray, pray.’ (P8, female, 23 years old)

## Theme 2: Attachment to baby

Being attached to the baby means loving to spend as much time with the baby as possible and at all times. Participants mentioned that they wanted to be with their babies at all instances, while they were at the hospital. One said:

‘Spending time with him, spending time with my baby, then I’m not alone and … yes it’s basically all, [*laughing*] because that’s all I can do in this place.’ (P2, female, 19 years old)

Similarly, another participant mentioned that being with her baby was the most important thing for her to do in order to cope. This participant also mentioned that to be with her baby all the times and get ready for discharge and her going home was her ultimate goal:

‘I just want my baby with me and want him to grow up and let us go home and that my … I will feel very glad [*laughed*], really, really.’ (P5, female, 29 years old)

Two subthemes emerged under the theme of attachment with baby which were bonding with baby and seeing the baby.

### Subtheme 1: Bonding with baby and seeing the baby

Frequent contact of participants with their babies helped them to interact and bond by kissing, playing, cuddling, touching, feeling and seeing their babies. This was illustrated by the participant who noted as follows:

‘Only for touching, if I go to him, I take him out of the incubator, put him to my breast, hold him to my arms, play with him, cuddle him, kiss him, and that attraction is getting me closer to him.’ (P1, female, 43 years old)

Participants mentioned that seeing baby, coupled with positive information from the health care professionals specifically the doctor regarding specific aspects about her baby, allowed coping better with the challenges of preterm delivery. This was highlighted by the one participant who said:

‘When I see the baby and when the doctor tells me no it’s going good with baby, the weight is coming up and then I feel good.’ (P5 female, 29 years old).

### Subtheme 2: Evidence of life progress on babies and no regrets

Participants expressed being grateful for the lives of their babies as well as for being able to experience motherhood irrespective of the state at which their baby was born as one mentioned that:

‘Ok. Everything about her, the way she looks at me. That is stuff it keeps me going. That is why I am not regretting the way she was born small.’ (P11, female, 20 years old)

Likewise, another participant seeing her baby and observing new behaviour in the life of the baby gave her sufficient reason to attach more to her baby, making her feel good and cope well this participant said:

‘Because I see my baby’s alive, he’s crying, he’s open[*ed*] his eyes, I have my baby now, he’s crying and he’s … opened his eyes. I hold my baby in my hands.’ (P10, female, 20 years old)

Another participant mentioned that her baby’s life gave her enough reason to be strong after preterm delivery as she mentioned:

‘Because I thought like what if she passed away, what will I feel? She’s still breathing; she’s still alive, so why am I feeling that way? So that encouraged me.’ (P9, female, 24 years old)

## Theme 3: Acceptance of the situation

In this study, participants remained positive about their babies in the neonatal unit. Participants accepted the situation. Two subthemes emerged: perseverance in the situation and participants knowing their responsibility. Participants mentioned that acceptance of the situation helped them to cope because it reduced their stress levels as explained by participant 1:

‘Uuh! I can’t explain how I was feeling! But, the time I was pregnant I was happy because I want to see him. To myself I was feeling ok [*during pregnancy*], he’s gonna [*going to*] be like the other two. … [*thought*] I’m gonna deliver him and take him home, look after him, take care of him. But now, this happen[*ed*], he’s a premature baby, I’m the mother, I have to be next to him, I have to be there for him, I have to take care of him. That’s why I’m still in hospital.’ (P1, female, 43 years old)

Another participant mentioned that certain aspects demonstrated her love to her baby so much so that she was able to cope with preterm delivery:

‘It wasn’t easy, yeah, but what keeps me going is the love I have for her. Ok, everything is about her. The way she looks at me. That is stuff that keep me going.’ (P11, female, 20 years old)

### Subtheme 1: Perseverance in the situation

Participants accepted the situation. A mother mentioned that accepting the situation was the best way to go about preterm delivery, as illustrated by the following quote:

‘It’s hard but there’s nothing I can do. I must stay strong for what I’m going through, because if I stress it’s also not gonna be well.’ (P4, female, 23 years old)

### Subtheme 2: Participants knowing their responsibility

Understanding responsibility allowed participants to cope better and remain beside the preterm baby. Mothers in this study preferred to stay at the hospital because they understood that there was no one else who could be in the position or bear the pain they could bear for their babies. One mentioned that:

‘The time I was pregnant I was happy because I want to see him. I, I, to myself I was feeling, Ok, fine, he’s gonna be like the other two. I’m gonna deliver him and take him home, look after him, take care of him. But now, this happen[*ed*], he’s a premature baby, I’m the mother, I have to be next to him, I have to be there for him, I have to take care of him. That’s why I’m still in hospital.’ (P1, female, 43 years old)

## Discussion

Findings of this study provided understanding of the coping strategies employed by mothers of preterm babies when admitted to a neonatal care unit. The study revealed that mothers of preterm babies used praying, attachment to baby and acceptance of the situation as coping strategies.

### Praying as a coping strategy

The study revealed that believing in the supernatural God can be used as one of the coping mechanisms at the time of difficulties. Mothers with preterm babies prayed to express thankfulness to God for giving them a baby, as well as requesting grace and strength to take them through such hopeless experiences for their premature babies. This is consistent with the results of a study reported by Arzani et al. (2015), who found that prayer was the most important strategy that mothers of preterm babies used to cope. Participants asked God to intercede and provide good health to their preterm babies. The majority of these participants relied on God’s mercy; hence, this was reflected in the religious background of the participants. Every participant expressed that they were either Christian or Muslim. This explained why they all depended on God to relieve them of the trauma they were going through. This is also in accordance with the study by Wachholtz, Sambamthoori and Morgantown (2013), which found that dependence on prayer by individuals who are facing a stressful event increased from the year 2002 and 2007.

Participants in this study trusted prayer as they felt that prayer gave them confidence, hope and certainty which diminished sadness, shock, mixed feelings, fear and deceit experienced because of their baby’s condition. This is in line with a study by Anderzen-Carlsson, Lamy and Eriksson (2014) who found that religious involvement has a strong emotional or mental influence on distress.

The theme of praying was supported by the concept of religion in the Brief COPE model. The Brief COPE model explains that participants continuously prayed and meditated as they also found comfort in their religion and spiritual beliefs as part of support coping (Carver 1997; Monzani et al. 2015). Prayer was put into practice by the participants as they called on God’s name at all times and expressed faith as they believed that God could help them keep their babies alive. Participants of this study also expressed faith that God could increase their strength to care for their babies, as well as overcome the stress caused by preterm delivery. It can be mentioned that participants of this study demonstrated personal prayers for thanksgiving and for God to relieve them of stress as well as healing their babies (Carvalho et al. 2014).

### Attachment to baby

Participants in this study found that attachment to their babies was through mother–baby bonding and seeing their babies frequently as well as evidence of life, progress on babies and no regrets. The attachment to a baby differs from mother to mother. For preterm babies, mother–baby bonding is usually interrupted by the baby’s admission to a neonatal care unit, and this separation can affect the mother’s attachment to the baby (Ncube, Barlow & Mayers 2016). Some mothers would prefer to stay away, because of fear and uncertainty, others would prefer seeing and touching their babies as well as looking after the treatment their babies receive from health care professionals. In this study, attachment was very important to mothers as these mothers demonstrated preference to remain beside their babies. This research revealed that closeness with the newborn has enormous benefits for the mothers’ coping strategies (Anderzen-Carlsson et al. 2014). Flacking et al. (2012) mentioned that attachment of mothers to their baby increases and secures mother–infant bonding. Bonding also augments mothers’ confidence and competence in the care of their preterm babies. This reduces chances of high levels of stress and anxiety as well as prevents depression in participants (Wu et al. 2013). Participants in this study experienced attachment to their babies by touching, kissing, playing with and cuddling their babies. These participants experienced joy and happiness, which helped to reduce fear in them as well as prevent rejection of their preterm babies; this is confirmed by a study which found that attachment of mother to baby leads to well-being of the mother as well as better growth and development in the preterm baby (Anderzen-Carlsson et al. 2014).

With respect to seeing the baby, mothers were happy to view and remain close with their babies. It gives them hope, seeing that their babies were merely having signs of life and having enough assistance from their nurses who were tasked with the responsibility of taking care of the babies in the neonatal care unit. Again, the mothers were approached by doctors and other health care professionals to give feedback on the progress of their babies. For these reasons, mothers expressed no regrets on having given birth to preterm babies. They rather looked forward to having their babies discharged home in a healthy condition.

One participant requested for counselling sessions to be offered to the mothers admitted in the neonatal care unit. It could also be a result of the fact that the participants were very lonely at the hospital and lacked intimate conversation sessions. Hence, some mothers requested that discussion sessions be offered at the hospital. Thus, because of unavailable counselling support and discussion sessions to distract mothers admitted in hospital from stressful moments, this mother preferred to stay away from her baby, without knowing the effects on both her baby and herself. Smith et al. (2012) found in a study that mothers of preterm babies explained that they coped better when they stayed away from the NICU and the hospital environment where the baby was admitted. Mother-infant bonding is a crucial factor for child development as well as maternal health (Rossen et al. 2016). Most parents tend to feel comfortable, relaxed and rested when they are with their children and vice versa. This is important as it contributes to well-being of the child or parent. Therefore, mothers who stay away from their babies because of one reason or another should be encouraged to bond with their babies.

### Acceptance of the situation

The participants in this research expressed that understanding their responsibility as mothers and persevering with the conditions imposed by preterm delivery enabled them to accept the situation. Thus, accepting the situation helped them to feel better, thereby enhancing positive coping. These participants persevered under every circumstance and stayed in the hospital with their preterm babies, not abandoning them there with the health care providers (HCPs). This is in accordance with the results of a study by Arzani et al. (2015), who explored mothers’ strategies regarding the birth of a premature baby and found that patience and strength were coping strategies used by mothers who delivered preterm babies. They also mentioned that patience and strength revived in mothers who took part in their study only after mothers realised the instincts of their motherhood rule. Participants mentioned that they were bored in hospital and had preferred to go in and out of hospital. Some mentioned that not having discussion groups, prayer sessions and talk sessions had increased their eagerness to leave their babies and seek distraction outside the hospital environment. It is proven in research by Smith et al. (2012) that mothers of preterm babies coped better when they stayed away from their babies who were admitted in a neonatal care unit. However, participants in this study remained in the hospital, in the neonatal care unit, to bond with their babies. This is because they understood their responsibilities in the lives of their babies. Acceptance of the situation was matched to the concept of acceptance in the Brief COPE model by Carver (1997). This means that participants tend to accept the realism of the situation, and accept living with the stressful situation, which enables coping (Carver 1997). According to literature, it is clearly stated that the cause of preterm labour and delivery is unknown (Shapiro-Mendoza & Lackritz 2012). Therefore, mothers who deliver preterm babies should be prepared in order to be ready to accept the event of preterm delivery and hospital admission. This will enable them to cope better than when it happens when the mothers are unaware of the expectations (Gebre, Gebremariam & Abebe 2015). It becomes the responsibility of the HCPs to educate mothers during antenatal periods to educate and prepare mothers for unforeseen circumstances such as preterm labour, delivery and admission. This is noted by a researcher (Gebre et al. 2015).

## Limitations of the study

This study was conducted at a single public hospital; the generalisability of the findings is limited and there was possibly missing relevant information from mothers of preterm babies in other hospitals. The scope of this study was focused only on the mothers whose preterm babies were under the support of the hospital doctors and nurses, but excluded mothers whose preterm babies were discharged or not admitted to the hospital, and this could be a selection bias to the study sample.

## Recommendations

Health care professionals understand more about the needs of mothers with preterm babies in order to support them better to cope with the stressful situation and improve the well-being of the mothers as well as their preterm babies. It is recommended that mothers with preterm babies need professional counselling support, while they are in the hospital and health care professionals to assist mothers of preterm babies to keep up with their personal, emotional and spiritual well-being after preterm delivery. The initial orientation about the environment and situation given to mothers of preterm babies on their immediate arrival to the neonatal care unit in the hospital is crucial for mothers to cope with the stressful situation. The research was conducted at one research site as the intention was not to generalise the results but to have a deeper understanding about the coping strategies of mothers with preterm babies. However, the researcher recommends a bigger scale study to be conducted on partners’ support and on mothers who have no social support structure for possible generalisation and policy impact.

## Conclusion

This research was conducted aiming to yield answers that were based on the coping strategies of mothers of preterm babies. Premature delivery is a stressful event and demands a lot of energy from the affected mothers. Despite the small sample of this study, results indicate that mothers of preterm babies have different strategies to manage the stressful event of preterm delivery. This study yields important information that could be considered when managing mothers who are affected by preterm delivery for better maternal and child care delivery. More research is required in this field for possible generalisation as well as understanding the support structures in place for mothers who deliver preterm babies.
